# Hereditary renal cell carcinoma syndromes: diagnosis, surveillance and management

**DOI:** 10.1007/s00345-018-2288-5

**Published:** 2018-04-21

**Authors:** Eamonn R. Maher

**Affiliations:** Department of Medical Genetics, University of Cambridge and NIHR Cambridge Biomedical Research Centre and Cancer Research UK Cambridge Cancer Centre, Cambridge, CB2 0QQ UK

**Keywords:** Genetics, von Hippel–Lindau review, Familial syndromic renal cancer

## Abstract

**Purpose:**

Genetic factors have been implicated in the pathogenesis of renal cell carcinoma (RCC), with around 3% of cases having a family history. A greater knowledge of the genetics of inherited RCC has the potential to translate into novel therapeutic targets for sporadic RCC.

**Methods:**

A literature review was performed summarising the current knowledge on hereditary RCC diagnosis, surveillance and management.

**Results:**

Familial RCC is usually inherited in an autosomal dominant manner, although inherited RCC may present without a relevant family history. A number of familial RCC syndromes have been identified. Familial non-syndromic RCC is suspected when ≥ 2 relatives are affected in the absence of syndromic features, although clear diagnostic criteria are lacking. Young age at onset and bilateral/multicentric tumours are recognised characteristics which should prompt molecular genetic analysis. Surveillance in individuals at risk of inherited RCC aims to prevent morbidity and mortality via early detection of tumours. Though screening and management guidelines for some inherited RCC syndromes (e.g. von Hippel–Lindau disease, Birt–Hogg–Dube syndrome, hereditary leiomyomatosis) are well defined for rare cause of inherited RCC (e.g. germline *BAP1* mutations), there is limited information regarding the lifetime RCC risks and the most appropriate screening modalities.

**Conclusion:**

Increasing knowledge of the natural history and genetic basis has led to characterisation and tailored management of hereditary RCC syndromes. International data sharing of inherited RCC gene variant information may enable evidence-based improvements in the diagnosis, surveillance protocols and management of these rare conditions.

## Introduction

**Fig. 1:**
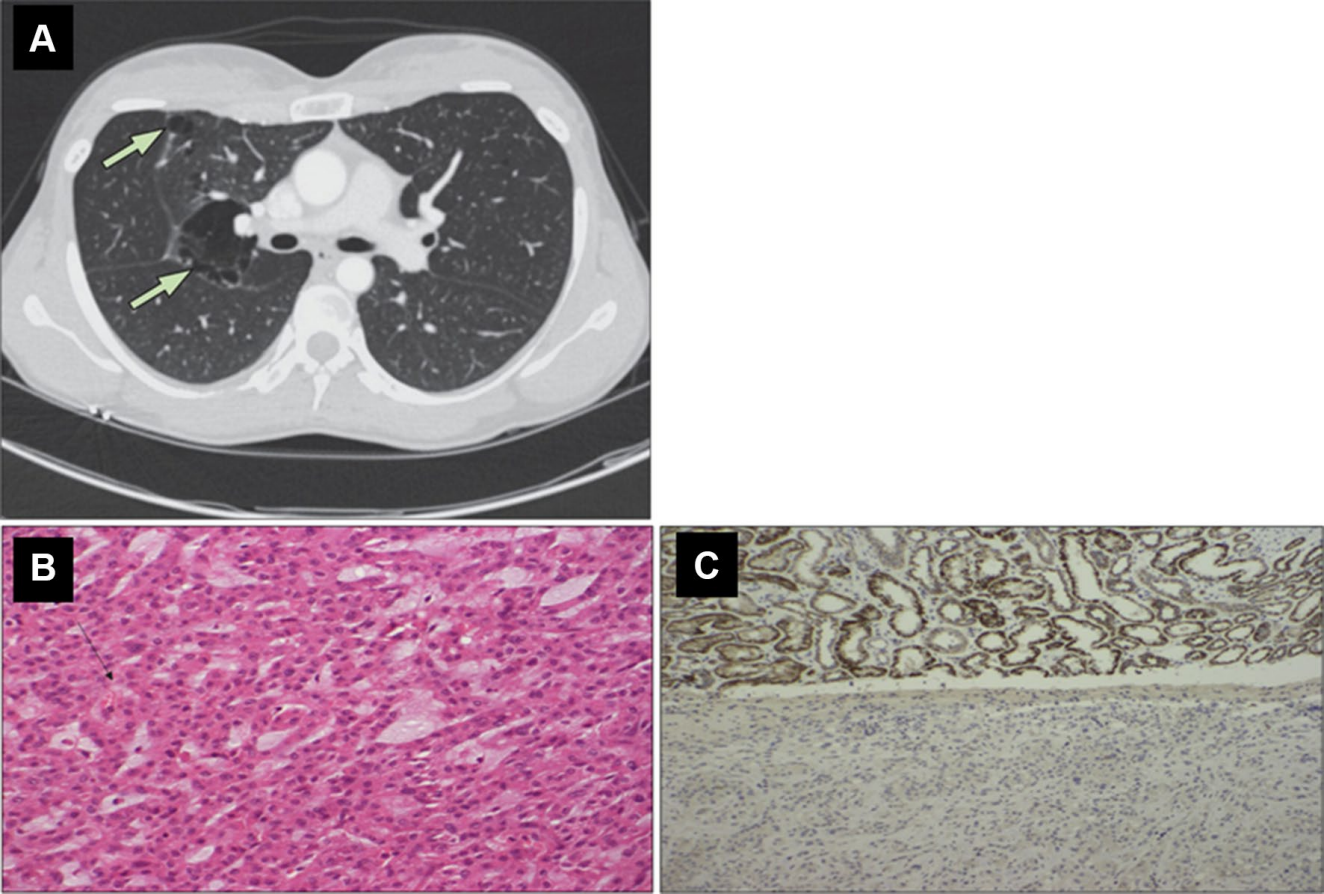
Examples of radiological, histological and immunohistochemical features that might suggest an inherited predisposition to renal cell carcinoma. Upper panel: **a** high-resolution CT thorax showing multiple basal cysts in a patient with Birt–Hogg–Dube syndrome (reprinted with permission from [52]). Lower panel: **b** the H + E-stained histological appearance of an SDHB-deficient RCC. There is evidence of intracytoplasmic vacuoles marked by the black arrow. **c** Loss of SDHB protein expression on immunostaining of the RCC tumour in the lower part of the image, with SDHB staining present in the adjacent normal renal tissue visible in the upper image. (Reprinted with permission from [39])

In addition to smoking, obesity and hypertension, genetic factors have been implicated in the pathogenesis of renal cell carcinoma (RCC) with around 3% of cases having a family history [1]. Familial RCC is usually inherited in an autosomal dominant manner (though there may be incomplete penetrance). However, patients harbouring a mutation in a gene predisposing to RCC do not necessarily have a family history of RCC (the mutation may have arisen de novo in the proband or the mutation may be non-penetrant in a carrier parent. If there is no family history, hereditary RCC might be suspected by the presence of bilateral/multicentric or young onset RCC and then confirmed by molecular genetic analysis. In addition, the presence of additional (non-RCC) clinical features in the proband or close relatives might suggest a specific multisystem hereditary RCC syndrome [e.g. pulmonary cysts in Birt–Hogg–Dube syndrome (see Fig. 1), cerebellar haemangioblastomas in von Hippel–Lindau disease; cutaneous or uterine leiomyomas in HLRCC, etc. (see later)]. Histopathological features or immunohistochemical investigations may also guide molecular genetic investigations (Table 1 and Fig. 1).

**Table 1:** Overview of major hereditary renal cell cancer syndromes. Adapted from Menko and Maher [1]

Syndrome	Inheritance	Gene	Estimated RCC risk	Renal tumour histological subtypes	Functional consequences of mutation
Von Hippel–Lindau disease	AD	*VHL*	70%	Clear cell RCC	Activation of hypoxic response pathways
Birt–Hogg–Dubé syndrome	AD	*FLCN*	25%	Various, but hybrid chromophobe/oncocytic RCC typical	Activation of the mTOR pathway
Hereditary type 1 papillary RCC	AD	*MET*	Increased	Papillary type 1 RCC	Activation of MET signalling pathway
Hereditary leiomyomatosis and renal cell cancer	AD	*FH*	15%	Papillary type 2 RCC	Activation of hypoxic response pathways Epigenetic changes (e.g. DNA methylation)
Succinate dehydrogenase subunit-related RCC	AD	*SDHB* *SDHD* ^a^ *SDHC* *SDHA*	Highest risk (up to 10–15% with SDHB	Various types, but specific features recognised	Activation of hypoxic response pathways Epigenetic changes (e.g. DNA methylation)
Chromosome 3 translocations	Chromosomal	Chromosome 3	Increased (up to 70%)	Clear cell RCC	Loss of translocated chromosome 3p and somatic mutation of VHL leads to activation of hypoxic response pathways
*PTEN* hamartoma tumour syndrome	AD	*PTEN*	5–35%	Mostly papillary RCC	Activation of phosphoinositide 3-kinase (PI3K) signalling pathway
Hereditary *BAP1* tumour syndrome	AD	*BAP1*	Increased	Clear cell	BAP1 inactivation associated with altered chromatin architecture, DNA damage response and cell cycle regulation

*AD* autosomal dominant, *RCC* renal cel carcinoma

^a^Inheritance is characterised by maternal imprinting

## Methods

A non-systematic literature search was conducted using Medline, updated to December 2017. The reference lists of selected manuscripts were checked manually for eligible articles. The most relevant articles summarising existing knowledge on hereditary RCC syndromes, including diagnosis, management and surveillance, were selected for this review.

## Results

### Major inherited forms of RCC

#### von Hippel–Lindau disease

This rare autosomal dominantly inherited disorder has an incidence of approximately 1 in 30,000 and is caused by constitutional mutations in the *VHL* tumour suppressor gene (TSG) [2, 3]. In most cases, the lifetime risks of retinal and central nervous haemangioblastomas and clear cell RCC are over 70% each [4, 5]. Less frequent tumours include phaeochromocytoma/paraganglioma (approximately 20% of cases), pancreatic neuroendocrine tumours (approximately 10%) and endolymphatic sac tumours (approximately 5–10%). Multiple visceral cysts (renal, pancreatic and epididymal) are common and may help indicate the diagnosis. More than 95% of patients with VHL disease will have a detectable VHL gene mutation and well-defined genotype–phenotype mean that the nature of the mutation may predict likely tumour risks (e.g. risk of phaeochromocytoma is small with truncating mutations and exonic deletions) [3, 5]. The VHL gene product has a critical role in regulating the hypoxic gene response through stability of the α-subunits of the HIF-1 and HIF-2 transcription factors [6–8]. This knowledge provides the rationale for the use of antiangiogenic tyrosine kinase inhibitors (e.g. sunitinib/sorafenib) in sporadic RCC, as most clear cell RCC have somatic mutations in the *VHL* TSG [3]. Patients with VHL disease and asymptomatic family members found to carry the familial mutation are screened annually to detect asymptomatic tumours [full details of the surveillance programmes are published elsewhere (10, 11), but generally renal screening is by annual MRI from age 16 years] and enable early intervention (small RCC are usually removed when they reach 3 cm diameter) [9–11].

#### Birt–Hogg–Dubé syndrome

Birt–Hogg–Dubé (BHD) syndrome is characterised by an autosomal dominantly inherited predisposition to multiple fibrofolliculomas (characteristically on the face), lung cysts and pneumothorax, renal cell carcinoma and possibly colorectal tumours [12, 13]. The risk of RCC in BHD syndrome is significantly less than that in VHL disease (approximately 25%), but annual renal surveillance (usually by MRI or renal ultrasonography if MRI is unavailable or not tolerated) is offered to patients and mutation carriers from age 20 years [13]. Though the characteristic RCC histology contains chromophobe and oncocytic elements, other subtypes, including clear cell RCC, are well described [14, 15]. Germline inactivating mutations in the *FLCN* TSG can cause both BHD syndrome and non-syndromic familial pneumothorax. The function of the *FLCN* gene product has not been fully elucidated; however, inactivation leads to activation of the mTOR pathway [16, 17].

#### Hereditary leiomyomatosis and renal cell cancer

The presence of multiple cutaneous leiomyomas in a patient with RCC suggests a diagnosis of hereditary leiomyomatosis and renal cell cancer (HLRCC). This very rare disorder (incidence approximately 1 in 200,000) is caused by inactivating mutations in the *FH* gene which encodes fumarate hydratase, a key component of the tricarboxylic acid (Krebs) cycle [18]. Affected females may present with early-onset multiple uterine leiomyomas (fibroids) and *FH* mutations are a rare cause of inherited phaeochromocytoma/paraganglioma [19–21]. RCC in HLRCC is typically classified as Type 2 papillary RCC or collecting duct RCC [22]. Though the lifetime risk of RCC in this condition is around 15%, it is typically an aggressive early metastatic tumour that can occur at a young age (mean 41 years, earliest report at 11 years) and therefore annual surveillance (by MRI as tumours may not be visualised by ultrasonography) is offered [19].

#### Succinate dehydrogenase-related RCC

Succinate dehydrogenase (SDH) is a tetrameric enzyme (encoded by the *SDHA*, *SDHB*, *SDHC* and *SDHD* genes) that is upstream of fumarate hydratase in the tricarboxylic acid (Krebs) cycle. Germline mutations in these *SDHx* were initially described in association with phaeochromocytoma/paraganglioma and head and neck paraganglioma (HNPGL), but the tumour spectrum has since expanded to include gastrointestinal stromal tumours, pituitary tumours and RCC [23–27]. A variety of histopathological subtypes have been described in *SDHx*-associated RCC, but recently a distinctive histopathology that should prompt molecular genetic investigations has been defined [28]. Though RCC has been associated with mutations in each of the subunits, the most commonly associated gene is *SDHB*. Germline mutations in *SDHB* may present with a familial RCC-only phenotype [26]. The lifetime risk of RCC in SDHB mutation carriers is not well defined, but likely less than 10–15%; however, annual or biannual renal surveillance by MRI can be combined with screening for phaeochromocytoma/paraganglioma (which commences in older children).

#### Hereditary papillary RCC

Activating mutations in the *MET* proto-oncogene predispose to Type 1 hereditary papillary RCC (HPRC) [29]. This is an extremely rare disorder that is inherited as an autosomal dominant trait with incomplete penetrance. Annual renal surveillance by MRI should be offered to patients and at risk relatives, but if the patient presents with metastatic RCC then treatment with a Met-inhibitor can be considered [30].

#### Hereditary BAP1-associated RCC

Following reports of germline *BAP1* mutations in familial uveal melanoma, cutaneous melanoma and mesothelioma [31, 32], it was recognised that RCCs are also part of the *BAP1* tumour syndrome spectrum [33, 34]. Though germline *BAP1* mutations have been described in patients with familial RCC and no other *BAP1*-related tumours, such cases are rare and *BAP1* mutation analysis is not yet performed in all cases of inherited RCC. There are no generally agreed surveillance protocols for *BAP1* mutation carriers, but, as with other inherited cancer predisposition syndromes, repeated irradiation should be avoided and therefore surveillance should be by MRI rather than CT scanning.

#### Constitutional chromosome 3 translocations

Constitutional chromosome 3 translocations are a rare, but well-validated cause of familial RCC. In patients with familial RCC, the detection of a constitutional chromosome 3 translocation is likely to be relevant (though the translocation breakpoints are variable), but if a chromosome 3 translocation is detected for another reason (e.g. prenatal diagnosis) and there is no personal or family history of RCC then the risk of RCC is very small [35].

#### Other rare conditions

A variety of other rare causes of RCC predisposition have been described. There has been increasing recognition of a significant risk of RCC in patients with Cowden/PTEN hamartoma tumour syndrome, but germline *PTEN* mutations are very rare in patients with non-syndromic inherited RCC [36]. Tuberose sclerosis may be associated with early-onset RCC, but RCC is rare in this condition and renal lesions are most commonly angiomyolipomas [37]. Germline *VHL, SDHx* and *FH* mutations may predispose to phaeochromocytoma/paraganglioma and RCC, and on rare occasions renal tumours have been reported in association with mutations in the phaeochromocytoma genes *TMEM127* and *MAX* [38, 39]. Germline mutations in *CDC73* are associated with hyperparathyroidism-jaw tumour syndrome, a very rare disorder that has been associated with Wilms tumour and, on one occasion, papillary RCC [40].

#### Familial non-syndromic RCC

There are no clear diagnostic criteria for this disorder, but broadly this condition is suspected when two or more relatives have RCC and there are no features to suggest an underlying “syndromic cause” (Table 1). The presence of early-onset tumours and/or multiple/bilateral tumours should further increase suspicion. Despite the absence of syndromic features, germline mutations in *SDHB* and *FLCN* are not uncommon in such cases and molecular genetic testing for an RCC gene panel [e.g. *FLCN*, *FH*, *MET*, *SDHB*, *VHL* (± *BAP1*)] and cytogenetic analysis is generally performed in suspected cases. However, in most cases a genetic cause is not identified. Some familial cases may result from chance or shared environmental factors or polygenic inheritance (genome-wide association studies have identified RCC susceptibility loci), but it is highly suspected that additional RCC predisposition genes remain to be identified [41, 42]. Familial non-syndromic RCC without an identifiable genetic cause is likely to be genetically heterogeneous and clinical studies suggest that autosomal dominant inheritance (in inherited cases) is the most likely form of transmission [15]. Germline *SDHB* and *FLCN* mutations have also been described in non-syndromic early-onset or bilateral RCC cases with no family history [15, 25].

### Diagnosis of familial RCC

Young age at onset and bilateral/multicentric tumours are well recognised features of inherited syndromic RCC and often present in non-syndromic RCC, and these are therefore indications for genetic analysis [45]. However, testing of individuals at low prior risk for a mutation can lead to diagnostic uncertainties from the identification of rare variants of uncertain significance (VUSs). In cases of inherited RCC with syndromic features, molecular genetic testing can usually be expected to unequivocally confirm the diagnosis, particularly for well-characterised genes such as *VHL* in which a wide range of germline mutations have been described and VUSs are relatively infrequent. For less well studied genes such as *BAP1* variant, interpretation can be more challenging. Therefore, as suggested for diagnostic testing of hereditary phaeochromocytoma and paraganglioma, the establishment of agreed gene panels and curated databases of inherited RCC-associated gene variants would facilitate expert genetic testing [46]. Another issue to be addressed is which groups of patients should be offered testing. The mean age at diagnosis of symptomatic RCC in VHL disease was around 45 years compared to an average age of > 60 years in sporadic cases [43], but there is not an agreed age threshold at which earlier-onset cases should be tested for RCC predisposition mutations. Schuh et al. [44] suggested that patients with sporadic RCC aged 46 years or younger should trigger consideration for germline mutation testing. However, the germline mutation detection rate will be low and for those that test negative the residual risk of an underlying genetic cause is unclear and so other centres have a lower threshold for testing (e.g. age 40 years). The low mutation detection rate in familial non-syndromic RCC suggests that there are further inherited RCC genes to be identified and achieving a comprehensive picture of the molecular architecture of inherited RCC is necessary for the development of optimal evidence-based guidelines for the molecular investigation of potential inherited RCC.

### Management and surveillance of inherited RCC

Over the past 25 years, the identification and surveillance of large numbers of *VHL* mutation carriers has led to a broad consensus to how they should be investigated and managed. In particular, a consensus has developed that small (< 3 cm) screen-detected tumours should be managed by active surveillance and then nephron-sparing surgery performed when a solid lesion reaches 3 cm in diameter [47]. As an alternative to partial nephrectomy percutaneous radiofrequency ablation has been used to treat small renal lesions in VHL disease [51]. In general, the approach to the management of RCCs identified through surveillance in VHL disease has been extrapolated to individuals with Birt–Hogg–Dube syndrome with tumours followed by active surveillance until they reach a diameter of 3 cm and then nephron-sparing surgery is performed (alternatively, radiofrequency ablation may be used to treat smaller tumours) [13]. However, it is clear that the “3 cm rule” is not suitable for renal lesions in HLRCC, which can metastasise early and are not reliably detected by renal ultrasound. Consequently, individuals with germline *FH* mutations undergo annual MRI surveillance even though most will not develop a renal lesion and surgical intervention is indicated for small screen-detected lesions [19]. For individuals with a germline *BAP1* mutation, there is very limited information on the lifetime risks of RCC and the most appropriate screening modalities. International data sharing of inherited RCC gene variant information and multicentre collaboration to pool results of natural history and screening protocols for mutation carriers are required to enable evidence-based surveillance programmes to be designed. For example, *SDHx*-associated RCC can be aggressive implying that surveillance for early detection is important. However, the tumour risks in *SDHB* mutation carriers are significantly less than originally thought and so there is (as in *FH* mutation carriers) a tension between over-investigation and early detection. This might be addressed by the development of biomarkers to identify the subset of individuals who will develop RCC and should be targeted for screening and/or novel early detection strategies (such as circulating tumour DNA biomarkers).

In some patients, particularly those with no previous family history, the diagnosis of inherited RCC disorder is only made after presentation with metastatic disease. Additionally, in VHL disease patients may develop multiple central nervous system haemangioblastomas that are not amenable to surgical treatment because of their critical location. Hence, there is a need to develop effective medical therapies for such cases. Most of the known inherited RCC genes encode tumour suppressor genes and biallelic inactivation of the relevant inherited RCC gene is present in all tumour cells. Therefore, in addition to standard therapies for metastatic RCC, targeted therapies with agents that exploit the specific molecular pathways provides a rational approach to therapy [48, 49]. The concept of synthetic lethality-based interventions is particularly interesting for inherited RCC (and haemangioblastomas) in disorders such as VHL disease, because the kidneys of VHL patients can harbour hundreds of small “tumourlets” with biallelic VHL inactivation, some of which will give rise to RCC years later [50]. Hence, it could be hypothesised that ablation of such tumourlets by administration of a synthetically lethal compound to young adults with VHL disease might reduce the risk of RCC at a later age. The development of novel therapeutic approaches to inherited RCC will require a deeper knowledge of the normal function of inherited RCC gene products and the consequences of mutations in the relevant pathways. However, a likely outcome of such research would be the potential for translating the knowledge of the pathogenesis of inherited RCC into novel treatments for sporadic RCC (as exemplified in VHL disease and the involvement of *VHL* inactivation in sporadic clear cell RCC).

## Conclusion

A number of familial RCC syndromes and inherited non-syndromic RCC have been identified. The presence of bilateral/multicentric or young-onset RCC, syndromic and histopathological features should prompt and guide molecular genetic analysis. The principal strategy for preventing morbidity and mortality in individuals at risk of inherited RCC is detection of early stage tumours which can then be removed (e.g. HLRCC) or followed up to a safe size (e.g. 3 cm diameter in VHL disease and BHD syndrome) when they are removed or ablated. International data sharing of inherited RCC gene variant information may enable evidence-based improvements in the diagnosis and management of these rare conditions.
